# Changes in the Separation Properties of Aged PVDF Ultrafiltration Membranes During Long-Term Treatment of Car Wash Wastewater

**DOI:** 10.3390/membranes15030066

**Published:** 2025-02-20

**Authors:** Wirginia Tomczak, Marek Gryta, Piotr Woźniak, Monika Daniluk

**Affiliations:** 1 Faculty of Chemical Technology and Engineering, Bydgoszcz University of Science and Technology, 3 Seminaryjna Street, 85-326 Bydgoszcz, Poland; monika.daniluk@pbs.edu.pl; 2 Faculty of Chemical Technology and Engineering, West Pomeranian University of Technology in Szczecin, Piastów Ave. 42, 71-065 Szczecin, Poland; marek.gryta@zut.edu.pl (M.G.); piotr.wozniak@zut.edu.pl (P.W.)

**Keywords:** car wash wastewater, *Escherichia coli*, fouling, membrane aging, polyvinylidene fluoride membrane, separation properties, ultrafiltration

## Abstract

Car wash wastewater (CWW) is complex waste that may be effectively treated by the ultrafiltration (UF) process. However, one of the most important challenges in implementing this process on an industrial scale is the fouling phenomenon membrane aging. Indeed, these may lead to a reduction in UF performance possibly associated with a loss in integrity of the fouled/aged membrane. Therefore, the main aim of the current study was to provide a comprehensive investigation on the changes in the separation properties of aged FP100 ultrafiltration membranes made of polyvinylidene fluoride (PVDF) with respect to their application for long-term treatment of CWW. For this purpose, studies were conducted for new membranes and membranes previously used for over 5 years in a pilot plant. As a feed, solutions of dextran, solutions of model organism *Escherichia coli* and synthetic CWW were used. It has been found that PVDF membranes demonstrated poor stability when in frequent contact with chemicals periodically applied for membrane cleaning. Indeed, the aged membranes were characterised by the increased porosity. However, it is important to note that membranes aging had no significant impact on the permeate quality during the UF process of synthetic CWW. Indeed, the obtained permeate was characterised by the turbidity lower than 0.25 NTU. Likewise, with regard to the separation of *E. coli*, the aged PVDF membranes ensured the high process efficiency and over 99.99% bacterial retention. In the interest of the growing potential of PVDF membrane in CWW treatment, the results obtained in the current work complement the findings made in this field.

## 1. Introduction

Untreated wastewater from vehicle washes may have a negative impact, primarily on human and animal health and the environment. This is due to the fact that it contains several contaminants, such as oils, detergent, greases, and toxic metals [1,2,3]. It is worthy of note that one of the fundamental challenge in the treatment of car wash wastewater (CWW) with the use of conventional methods is the presence of *Escherichia coli* bacteria [4]. It is due to this fact that it is a public health issue [5,6,7,8,9,10]. It is evident from the available literature that *E. coli* is a major cause of urinary tract infection, enteritis, as well as other infections [7]. In addition, it may lead to meningitis, skin structure infections, myositis, and osteomyelitis [11].

Ultrafiltration (UF), a low-pressure membrane filtration process, is one of the most commonly used wastewater treatment processes. This is due to the fact that it provides low operation costs, facile operation, and high separation efficiency [12,13,14,15,16,17]. Consequently, in the past few years, there has been a particular research focus on the application of UF for the CWW separation, e.g., Refs. [18,19,20,21,22,23]. The selection of a suitable UF membrane is very crucial for promoting both high retention and permeate flux. Of particular note are polyvinylidene fluoride (PVDF), characterised by outstanding properties such as high mechanical strength, excellent thermal stability, and resistance; various organic solvents; and chemical agents such as acids, chlorine, and solvents [14,24]. Nevertheless, according to [25], the chemical stability does not apply to strong solutions of bases solutions, ketones, or esters. Moreover, due to hydrophobicity nature and low surface energy, PVDF membranes are prone to the fouling phenomenon due to organic feed components [13]. Fundamental properties of PVDF have been thoroughly presented and discussed in several excellent reviews [26,27,28,29,30,31,32].

As an example, performing a systematic review indicated that PVDF ultrafiltration membranes FP100 have been successfully used in several applications. Indeed, they have been used for the treatment of: (i) oil-in-water emulsion [33,34], (ii) car wash wastewater [20], (iii) metalworking fluids [35,36], and (iv) seawater [37] and bilge water [38,39].

Undoubtedly, the use of polymeric membranes on an industrial scale requires long-term efficiency and effectiveness of the process. Therefore, membrane fouling leading to flux decline is a great obstacle in the implementation of membrane technology [40,41]. Moreover, mitigation of severe fouling requires routine intensive and aggressive cleaning processes with the use of acidic and alkaline chemical agents [42,43]. Furthermore, membrane ageing may have serious consequences, such as modification of the physical–chemical and separation properties of the membrane as well as a loss of its integrity [44,45]. These phenomena may have a significant effect on the process performance and fouling behaviours. For this reason, the possibility of potential damage of the structure and properties of PVDF membranes during the long-term process should be considered.

An important point which should be noted is that although polymeric membranes dominate the wastewater industry [46,47,48,49,50,51], performing literature review allowed us to determine that the literature contains no experimental work investigating the changes in the separation properties of PVDF membranes during long-term application for the CWW treatment. This finding is in line with the indication pointed out by Zhang et al. [52] that literature focused on the properties and fouling behaviours of aged UF membranes are scarce. Moreover, several investigations regarding to the stability of PVDF membranes against sodium hypochlorite (NaOCl) solutions have been performed [53,54,55,56,57], while there is a significant lack of studies on the impact of sodium hydroxide (NaOH) solutions on the membranes separation properties.

Accordingly, to the best of the authors’ knowledge, this paper is one of the first attempts to provide a comprehensive investigation of the changes in the separation properties of the aged FP100 ultrafiltration membranes made of PVDF with respect to their application for the long-term treatment of CWW. For this purpose, the analysis was performed for new membranes and membranes previously used for over 5 years in a pilot plant and explored using NaOH solutions. Since UF membranes are known to be efficient for microorganism separation, in the current study, the membranes’ effectiveness was evaluated mainly by the separation degree of *E. coli* bacteria. In addition, to determine the membranes effectiveness in removing surfactants, chemical oxygen demand (COD) and turbidity, studies were also conducted using synthetic CWW as a feed.

## 2. Materials and Methods

### 2.1. Membranes

In the current work, the tubular FP100 membranes from PCI Membranes (Kostrzyn Wielkopolski, Poland) made of polyvinylidene fluoride (PVDF) were used. The membranes diameter and length were equal to 1.25 cm and 120 cm, respectively. The molecular weight cut-off (MWCO) declared by the manufacturer was 100 kDa. These membranes were successfully used to separate real car wash wastewater. However, daily membrane washing with alkaline cleaning solutions was required [20].

The results of UF separation tests were analysed for the three types of membrane samples differing in terms of operating conditions and time:

(i) “FP100”—the new membranes are rinsed several times with DI water (5 h),
(ii) “Rinsed”—chemical cleaning changes the membrane morphology [20,58]; thus, the new membranes which are subjected to preliminary degradation under the influence of aggressive cleaning agents. For this purpose, after dextran separation, membrane “FP100” was rinsed with DI water (5 h), alkaline P3 Ultrasil 11 solution (4 × 0.5 h), 0.5% H_3_PO_4_ (0.5 h), DI water (1 h) and then conditioned for 20 h with an alkaline (pH = 11.5) cleaning agent (“Insect”) and filtered (5 h/day, TMP = 0.1 MPa). The above-mentioned procedure corresponded to the impact of cleaning agents that would occur during more than 3 months of operation of the UF installation at a car wash [20].
(iii) “Pilot”—the FP100 membranes operated periodically for over 5 years in a UF pilot installation. The pilot installation was described in the previous work [33]. This installation was equipped with the PCI B1 tubular module equipped with 18 membrane tubes FP100 (total membrane area: 0.9 m^2^). The installation was used to filter water contaminated with machine oil (50–200 ppm), and for the last 2 months, synthetic car wash wastewater—which was a mixture of cleaning agents (active foam) and waxing used for car paint protection (“Hydrowax”)—was filtered. The composition of these cleaning agents was presented in the previously published study [21]. Due to fouling, the membranes in the pilot installation were systematically (every 5–7 days) washed using alkaline cleaning agents (P3 Ultrasil 11 or “Insect”), and during downtime, the installation was rinsed with the 0.25% sodium disulfite (Na_2_S_2_O_5_) solution for 30 min. As a result, the permeate flux was maintained at the level of 70–80% of the initial flux. However, as it was found, chemical washing also caused minor damage of the polyethersulfone (PES) membranes [21,58]. For this reason, the study investigated whether long-term use of FP100 membranes would deteriorate separation.

### 2.2. Experimental Set-Up

**Figure 1:**
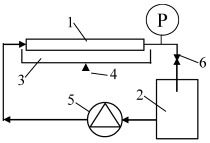
Experimental setup of the UF. 1—tubular membrane, 2—feed tank, 3—permeate tank, 4—balance, 5—pump, 6—valve, P—manometer.

The UF separation tests were conducted with the use of installation, as shown in Figure 1. Membrane samples with lengths of 25 cm (area 98 cm^2^) were installed in the laboratory system. The installation was fed with a piston pump, obtaining a feed flow of 1 m/s. The initial volume of feed samples was 2 L. The experiments were performed under transmembrane pressure (TMP) equal to 0.1 MPa and ambient temperature (293–295 K).

Before investigating the bacteria separation, the UF installation was thoroughly washed. To protect against airborne contamination, the installation was installed in a fume hood, which was thoroughly washed and disinfected with the use of a 70% ethanol solution. In order to disinfect the UF installation, the “Insect” solution (pH = 11.5) was filtered for 5–6 h. Subsequently, the installation was rinsed with a P3 Ultrasil 11 solution (pH = 11.8) for 30 min and then rinsed several times with DI water (Ellix 3, Millipore, Burlington, MA, USA). Finally, the installation was rinsed with 10% isopropanol, which remained in the installation overnight. Immediately before the experimental tests, the installation was rinsed several times with DI water and irradiated with a UV lamp for 1–2 h.

Obviously, long-term use and chemical cleaning of membranes may damage their surface [21,58]. The changes in the separation degree were determined under TMP of 0.1 MPa with the use of dextran solutions.

### 2.3. Feed Solutions

In order to determine the separation properties of membranes, three types of the feed were used:

(i) Solutions of dextran (Polfa, Łódź, Poland) with a molecular weight within the range of 100 and 500 kDa. The solution concentration was equal to 0.5 g/L.
(ii) Solutions of model organism *Escherichia coli* K12 (ATCC 29425). The feed volume was 2 dm^3^. The initial bacterial concentration in the feed was approximately 2.1–3.5·10^6^ CFU/mL. Similar bacterial content was found in real car wash wastewater analysed in [59].
(iii) Synthetic CWW consisting of a mixture of 0.5 vol.% foaming agent solution Turbo Active Foam Green (EuroEcol, Łódź, Poland) and 0.2 vol.% Hydrowax (EuroEcol, Łódź, Poland), characteristics of which were presented in a previously published work [60]. The composition of CWW was the same as the composition of effluents generated during car washing and contained surfactants (789 mg/L anionic and 33 mg/L nonionic) and polymeric waxes. It was characterized by the COD = 2680 mg/L and tortuosity equal to 23 NTU.

### 2.4. Membranes Cleaning

For washing FP100 membranes contaminated with oily wastewaters, the manufacturer recommends using an alkaline agent P3 Ultrasil 11 (Suturamed, Szczecin, Poland), which was used in a 0.1 wt.% solution (pH = 11.8) to clean membranes in a pilot installation. Car wash owners are reluctant to use chemicals not intended for washing cars. For this reason, in order to eliminate the use of additional chemicals in the car wash system, a 0.5 vol% solution of “Insect” agents (EuroEcol, Łódź, Poland) was additionally used to wash the membranes. This agent is used for removing insects from car bodies, and similar to P3 Ultrasil 11, it contains NaOH, EDTA tetrasodium salt, and surfactants [33]. Deionized water (DI water) (Elix 3, Millipore, USA) was used to prepare solutions and rinse the UF system.

For SEM examination of skin layer damage, the membrane sample collected from the module was soaked for 10 min in concentrated HCl, which allowed for complete removal of the deposits from the membranes.

### 2.5. Analytical Methods

The Hach cuvette tests were used to determine the concentrations of surfactants (LCK 333—nonionic, LCK 432—anionic) and COD (LCK 1014).

The turbidity of the test solutions was determined using a portable turbidimeter, model 2100 AN IS (HACH, Loveland, CO, USA).

The surface of the membranes was observed using a Hitachi SU8000 Field Emission Scanning Electron Microscope (FESEM; Hitachi, Tokyo, Japan). For investigation, the sample was taken by cutting a 2 cm tubular membrane from the feed inlet side.

The concentration of dextrans was analysed using a high-performance liquid chromatograph (UlitiMate 3000, Dionex, Sunnyvale, CA, USA) with PolySep-GFC-P 4000 column (Phenomenex, Torrance, CA, USA).

The rejection efficiency R (%) was determined as follows:

(1) $$R=\frac{C_{F}-C_{P}}{C_{F}}\times 100\%$$

where C_P_ (mg/L) and C_F_ (mg/L) are the measured concentrations of the permeate and feed, respectively.

Bacterial cultures were carried out in Petri dishes. To determine the number of *Escherichia coli* cells, TTC lactose agar containing the selective agent Tergitol-7 (BioMaxima, Lublin, Poland) was poured onto the plates. The incubation process was carried out at 37 °C for 24 h. The number of colonies was counted using a semi-automatic colony counter LKB 2002 (POL-EKO-APARATURA, Wodzislaw Slaski, Poland). The number of bacteria was expressed in CFU/mL (colony-forming units). The results are the average of three inoculations.

## 3. Results and Discussion

### 3.1. Membranes Performance

**Figure 2:**
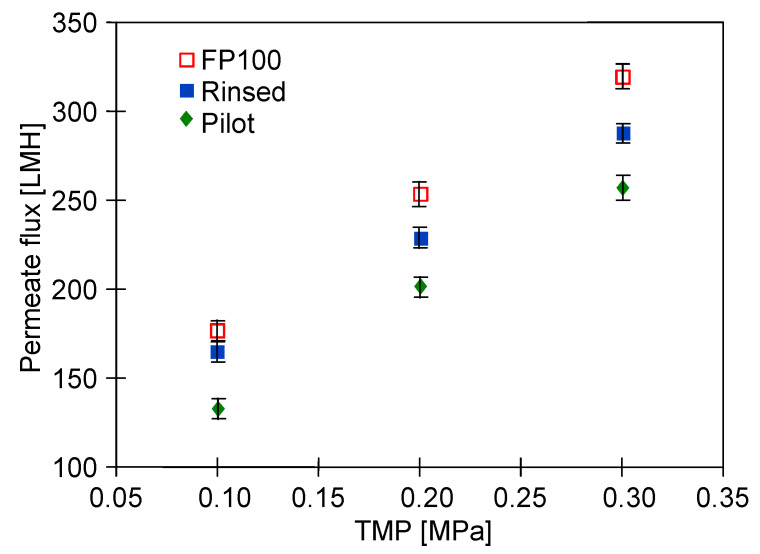
The maximum permeate flux as a function of TMP. Feed: DI water.

Figure 2 shows the maximum permeate flux determined for three types of membrane samples used in the present study (Section 2.1). It has been determined that for a TMP of 0.2 MPa, the maximum performance of the new membranes (“FP100”) was equal to about 252 LMH. However, it has been found that rinsing the membranes after dextran and bacterial separation with the use of P3 Ultrasil 11 solution followed by 20 h “Insect” filtration (‘Rinsed’) allowed us to achieve lower permeate flux. Indeed, for a TMP of 0.2 MPa, it was equal to about 225 LMH. It has been documented that these cleaning agents contain surfactants and NaOH, which effectively remove contaminants from membranes generated during UF car wash wastewater [20]. However, it is well known that long-term exposure to NaOH solutions on membranes causes swelling and degradation of polymer chains [61]. Such changes are also caused by filtration of “Insect” solution (pH = 11.5), which resulted in a slight decrease in permeate flux [58]. In addition, cleaning agents contain surfactants that enhance the removal of contaminants from membranes, nevertheless, they can also adsorb into the pores, which slightly reduces the membrane permeability [60]. On the other hand, as expected, the lowest performance was determined for the membranes collected from the pilot installation (“Pilot”). At the TMP mentioned above, a flux equal to about 200 LMH was noted. This can be explained by the fact that these aged membranes were characterized by the irreversible fouling caused by the long-term operation thoroughly discussed in the previously published papers [16,23].

Finally, it should be pointed out that for the membrane used, the permeate flux depended linearly on the applied TMP. For instance, for the new membrane, increasing the TMP from 0.1 to 0.2 and 0.3 MPa led to an increase in the permeate flux from about 170 LMH to 252 and 320 LMH, respectively. Obviously, the higher flux for higher TMP is supported by the greater hydrodynamic driving force toward the membranes [44,45]. In the literature, this phenomenon has been widely documented for various types of UF membranes [62,63,64,65].

**Figure 3:**
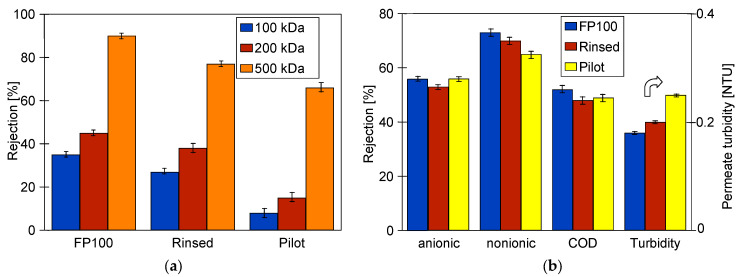
Separation degree of: (**a**) dextran; (**b**) surfactants (anionic and nonionic), COD, and turbidity for the membranes used. “Rinsed”—membranes used after 20 h of “Insect” solution filtration (pH = 11.5), “Pilot”—membranes used for 5 years for wastewater separation with cyclic chemical cleaning with alkaline cleaning agents.

In the next stage of the study, the retention degree of dextrans for the tested membranes was analysed (Figure 3a). It has been recognised that it can be presented in the following order: “FP100” ˃ “Rinsed” ˃ “Pilot”. Indeed, the new membranes (Figure 3a, “FP100”) ensured the highest retention degree for both dextrans, with MWCOs of 100 kDa, 200 kDa, and 500 kDa. In another study [58] a 100 kD polyethersulfone (PES) membrane retained 500 kDa dextran by 100%. The new FP100 had a lower rejection rate (92%). The studies of the membranes obtained from the manufacturer showed that their skin layer contained holes of 20–40 nm in size [20]. Such damage was also present in the samples tested in the current study, as shown in the SEM image in the Section 3.3. On the other hand, the membranes previously used for the pilot installation (Figure 3a, “Pilot”) were characterized by the lowest separation degree since it did not exceed 70%. It can be explained by the fact that these membranes were often cleaned with the use of chemical agents, and as a result of polymer degradation, the number of large pores increased (diameter up to 200 nm) [33]. Numerous instances of skin layer damage were also confirmed in this work (Section 3.3). Hence, it can be concluded that PVDF membranes demonstrated poor stability when in frequent contact with chemicals, such as alkaline cleaning agents. Surprisingly, it has been noted that the separation degree components of the synthetic wastewater for all the tested membranes were similar (Figure 3b). The noted high separation degree of surfactants, COD, and turbidity for the “Pilot” membrane can probably be explained by the accumulation of feed constituents on the membrane during the previously implemented processes. Finally, it should be noted that the membrane’s aging has no significant impact on the permeate quality. Indeed, all tested membranes provided a permeate characterised by turbidity lower than 0.25 NTU. The findings presented above demonstrate that all tested membranes applied for CWW separation ensured high permeate quality regardless of the contamination degree and aging time.

### 3.2. Membranes Exploitation

**Figure 4:**
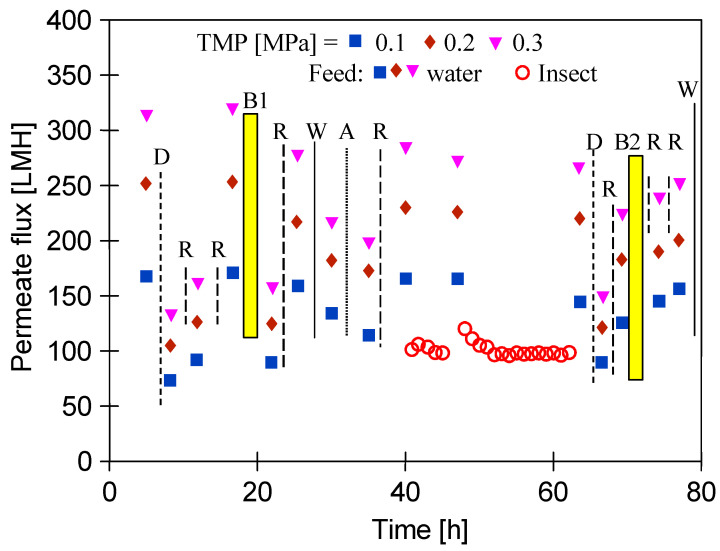
Changes in the maximum permeate flux during exploitation of the “FP100” membranes. Points: D—determination of dextran separation, R—washing with P3 Ultrasil 11 (pH = 11.8) 30 min, A—washing with 0.5% H_3_PO_4_ (30 min) and then rinsing with DI water. B1, B2—filtration of *E. coli* bacteria. W—wastewater separation. Insect—filtration with 0.5 vol.% Insect solution (pH = 11.5).

The measurement series carried out in this work were multi-stage. Undoubtedly, the conducted filtrations as well as the performed cleaning of the tested membranes had an influence on the changes in the maximum permeate flux. The noted changes during the exploitation of the new membranes (“FP100”) are presented in Figure 4. The flux decreased after dextran separation (Figure 3a) from 320 to 140 LMH (TMP = 0.3 MPa), and its recovery required two consecutive rinses of the membrane with P3 Ultrasil 11 solution (Figure 4, “R” up to 15 h). As a consequence, the flux increased to 325 LMH (TMP = 0.3 MPa).

After membrane washing and recovery of the initial permeate flux, filtration of *E. coli* bacteria was performed (Figure 4, “B1”), which led to a twofold decrease in the membrane’s performance. Therefore, it can be indicated that the membranes showed a fouling propensity, which can be attributed to their hydrophobic nature. Despite repeated alkaline cleaning, the permeate flux recovered was up to 275 LMH (TMP = 0.3 MPa). Then, separation of the wastewater was carried out (Figure 4, “W”), which resulted in a decrease in the flux to 220 LHM. The results of CWW separation are presented in Figure 3b (FP100). After acid and alkaline washing of the membrane (A and R, respectively) the permeate flux increased to 285 LHM. In the next step, in order to determine the chemical resistance of the PVDF membrane, the 0.5% “Insect” solution (pH = 11.5) was filtered for over 20 h. After that, the performed analysis of the dextran separation showed a slight deterioration of retention (Figure 3, “Rinsed”). This finding indicated that the NaOH interaction occurring during membranes washing and insect filtration could have caused the loosening of the skin layer of the used PVDF membranes. Such effects for PES membranes are clearly presented in the study [58]. This conclusion was also confirmed by the significant reduction in rejection degree for the CWW components (Figure 3b, “Rinsed”).

**Figure 5:**
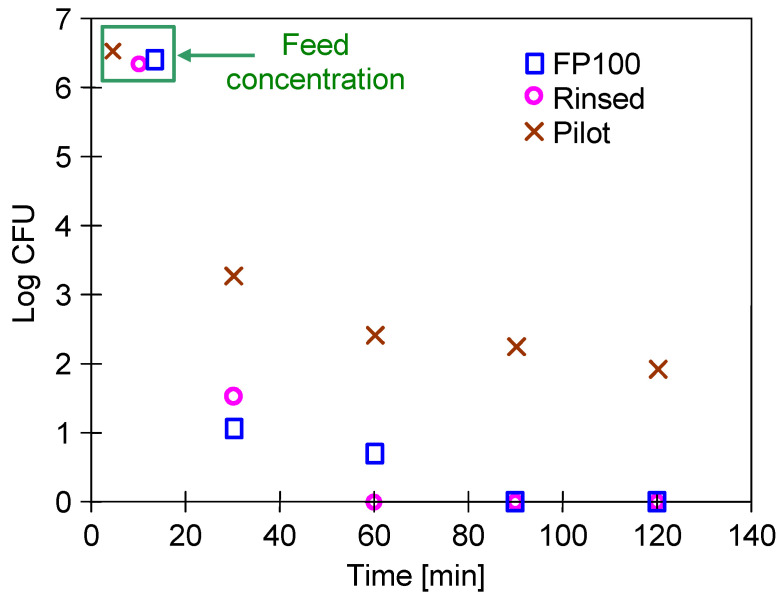
Bacterial separation using the FP100 membranes.

At the car wash, the washing fluids flow out of the nozzle at high pressure, which favours the formation of an aerosol. When washing cars, it is difficult to avoid inhaling such aerosol; therefore, the reused water should be microbiologically clean. In order to determine whether long-term exposure to NaOH solutions reduces the rejection degree, bacteria separation was performed again (Figure 4, “B2”). The results of the microbiological analysis are presented in Figure 5.

The obtained results showed that is possible to achieve complete bacterial separation for the new membranes (FP100) as well as for the “Rinsed” membrane. More precisely, the feed solutions “B1” and “B2” contained 2.1–3.5 · 10^6^ bacteria/mL. In the case of the feed “B1”, after 30 min of the UF process run, the permeate contained 6–12 bacteria/mL (Figure 5, ‘FP100’). Importantly, after 120 min, the obtained permeate was sterile. In turn, in the case of the feed B2 (Figure 5, “Rinsed”) after 30 min of UF process, there was 30–39 bacteria/mL in the permeate. However, after 60 min, the permeate was microbiologically clean. These findings indicate that bacterial rejection is influenced by both the membranes properties and the cake layer on their surface. *E. coli* is a rod-shaped and gram-negative bacteria which belongs to the Enterobacteriaceae family. Its cells are characterised as being 1–3 μm × 0.4–0.7 μm in size, 1 μm long, and 0.35 μm wide [66,67]. Hence, in general, these microorganisms are characterised as being much larger than the pores of UF membranes. For this reason, it has been assumed that the formation of small damages should not significantly decrease the separation degree for bacteria. Indeed, it was confirmed by results presented in Figure 5. From the view of the CWW treatment industry this finding is significant since effective fouling control allows to reduce the frequency of membrane replacement and is consistent with principles of resource efficiency [43].

**Figure 6:**
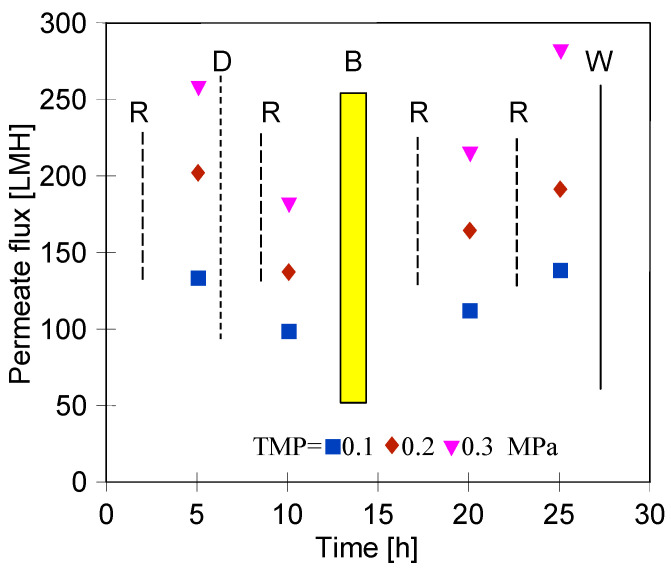
Changes in the maximum permeate flux during the exploitation of the “Pilot” membrane. Points: D—determination of dextran separation, R—washing with P3 Ultrasil 11 (pH = 11.8) 30 min, B—filtration of *E. coli* bacteria. W—wastewater separation.

It should be pointed out that results for the “Rinsed” membranes were obtained after several weeks of testing. However, it has been assumed that the separation properties of membranes deteriorate during longer periods of operation. To determine this, a membrane sample (“Pilot”) was taken for testing from a UF module used for 5 years. The changes in permeate flux are shown in Figure 6.

Despite 5 years of membrane exploitation, the obtained permeate flux was only slightly lower than the values obtained for new membranes (Figure 4). Although dextran rejection was significantly lower than those obtained for new membranes (Figure 3a, “Pilot”), the CWW separation results were only slightly less effective (Figure 3b). However, such results clearly indicated that membrane damage increases with the time of their exploitation. Due to them, the retention of bacteria also improved, and as a result, the permeate contained 218–390 bacteria/mL for a feed containing 3.3 × 10^6^/mL bacteria (Figure 5, “Pilot”).

### 3.3. Membranes Fouling

**Figure 7:**
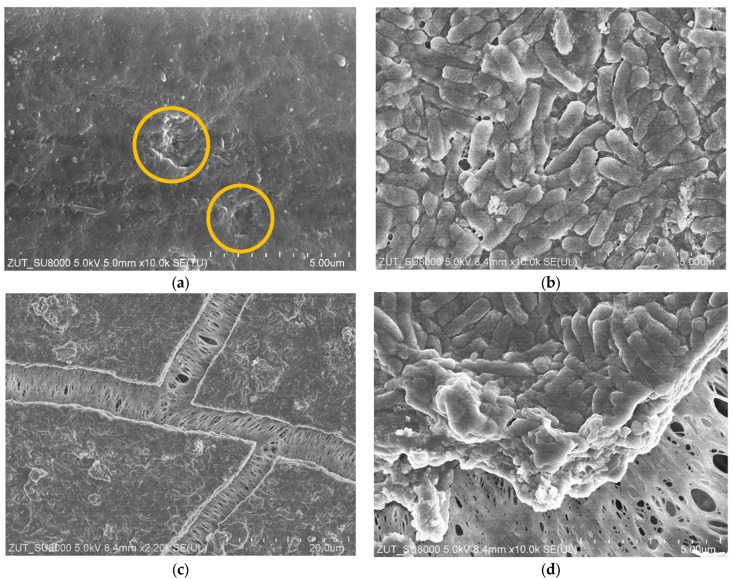
SEM images of the FP100 membrane: (**a**) FP100 new membrane; yellow circles—skin layer damages; (**b**) membrane surface covered with a layer of bacteria; (**c**) dried membrane—crushed and cracked sediment; (**d**) fracture boundary with a biofilm layer.

Notably, the research results presented and discussed above were explained by implementing SEM analyses. Indeed, after each filtration run, a sample of the membrane was analysed with the use of SEM. Firstly, the presence of bacteria in the permeate during the first 30 min of the UF process (Figure 5) can be explained by the little skin layer damage (Figure 7a, yellow circles). Secondly, a thick layer of bacterial cells formed on the membrane surface (Figure 7b) was the reason of the higher separation degree observed during the UF process run. The membrane sample with bacterial deposit was dried naturally, which caused it to crack. It should be pointed out that large pores (over 1 micrometer) were visible in the cracks (Figure 7c). Undoubtedly, they were created as a result of the stretching of the skin layer by the shrinking drying deposit. This finding confirmed that drying UF membranes causes damage to them. The image of fracture boundary with a biofilm layer allowed us to estimate the thickness of the biofilm to be over 2 μm (Figure 7d).

**Figure 8:**
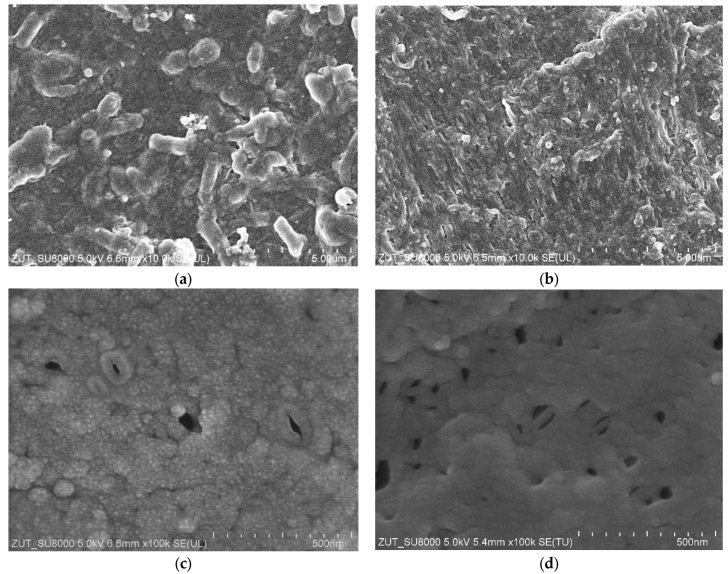
SEM images of the membranes: (**a**) FP100 membrane rinsed after UF of the feed “B2”; (**b**) biofilm residues on the surface FP100 membrane after it chemical cleaning; (**c**) FP100 membrane—deposit was removed by soaking membrane sample in concentrated HCl solution; (**d**) “Pilot” membrane after UF tests—deposits were removed from sample by soaking membrane in concentrated HCl solution.

After filtration of the feed “B2” (Figure 4) the membrane surface was again covered with a thick layer of biofilm (Figure 8a), which ensured a high separation degree (Figure 5, “Rinsed”). Despite several membrane-washing steps, the surface was not completely cleaned (Figure 8b). This aim was achieved after soaking the membrane sample in 30% HCl solution for 10 min (Figure 8c). In the places where the deposit was removed, there were no large pores visible in the sediment cracks (Figure 7c,d), which confirms that they were formed as a result of the deposit drying. However, the presence several holes about 100 nm in size was observed in the membrane. Such damage was almost absent in the pristine membranes (Figure 7a). This finding showed that routinely using of chemical agents (mainly P3 Ultrasil 11 and “Insect”) in order to restore the membrane’s performance (Figure 4) destroyed its skin layer structure. However, there were significantly more of them in the membrane used in a pilot installation, which was chemically cleaned much longer and more often (Figure 8d). These findings clearly demonstrated that regular cleaning of PVDF membranes with alkaline chemical agents leads to damage of the membranes skin layer. This resulted in a significant decrease in the rejection degree for the “Pilot” membrane compared to those for the “FP100” and “Rinsed” membranes (Figure 3 and Figure 5).

**Figure 9:**
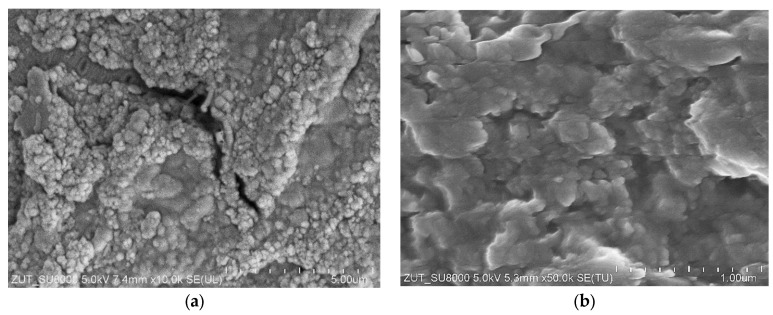
SEM images of the “Pilot” membrane: (**a**) membrane after 5 years of the exploitation; (**b**) membrane after rinsing with alkaline agents.

Overall, it has been found that although PVDF membranes are being exposed to alkaline solution and subjected to frequent chemical cleaning, a layer of deposit accumulated on the membranes surface during 5-year exploitation in a pilot installation (Figure 9a). As a result, the lowest values of the maximum permeate flux were obtained for this membrane sample (Figure 2). Surprisingly, additional alkaline washing of “Pilot” membranes carried out at the beginning of the presented studies slightly washed away and loosened the deposit (Figure 9b). This—in turn, with a greater number of membrane damages (Figure 8d)—favours the presence of bacteria in the permeate. However, this phenomenon occurred only to a small extent, since during ultrafiltration, the permeate contained 218–390 bacteria/mL for a feed containing 3.3 × 10^6^/mL bacteria (Figure 5, “Pilot”).

## 4. Conclusions

This paper has highlighted the separation properties of the aged FP100 ultrafiltration membranes made of PVDF used for the long-term separation of CWW. The membranes’ effectiveness has been evaluated by the separation degree of dextran, *E. coli* bacteria, and synthetic CWW. Results obtained in the current study clearly demonstrated that the aged membranes allowed for almost complete retention of bacteria present in a feed. It has been well proved that the separation is enhanced by the formation of a biofilm on the membrane surface. The intensive fouling phenomenon was reduced by cyclically cleaning of the membranes with alkaline solutions. Although after more than 5 years of membrane cleaning, the presence of numerous holes of up to 100 nm in size was found in the membrane skin, the separation degree was similar to that noted for new membranes. It can be explained by the fact that the biofilm layer formed during the UF process ensured the high process efficiency. Finally, the outcomes of this study provide information on the separation properties of aged PVDF membranes used for the long-term treatment of CWW.

## Data Availability

The original contributions presented in the study are included in the article; further inquiries can be directed to the corresponding author.
